# Hepatocyte Nuclear Factor-1α Increases Fibrinogen Gene Expression in Liver and Plasma Fibrinogen Concentration in Rats with Experimental Chronic Renal Failure

**DOI:** 10.3390/ijms24065733

**Published:** 2023-03-17

**Authors:** Elzbieta Sucajtys-Szulc, Alicja Debska-Slizien, Boleslaw Rutkowski, Ryszard Milczarek, Marek Szolkiewicz, Julian Swierczynski, Ryszard Tomasz Smolenski

**Affiliations:** 1 Department of Nephrology, Transplantology and Internal Medicine, Medical University of Gdansk, Smoluchowskiego 17, 80-214 Gdansk, Poland; 2 Department of Pharmaceutical Biochemistry, Medical University of Gdansk, Debinki 1, 80-211 Gdansk, Poland; 3 Department of Cardiology and Interventional Angiology, Kashubian Center for Heart and Vascular Diseases, Pomeranian Hospitals, Jagalskiego 10, 84-200 Wejherowo, Poland; 4 Department of Biochemistry, Medical University of Gdansk, Debinki 1, 80-211 Gdansk, Poland; 5 Institute of Nursing and Emergency Medical Services, State School of Higher Vocational Education in Koszalin, Lesna 1, 75-582 Koszalin, Poland

**Keywords:** hepatocyte nuclear factor 1α, fibrinogen, clofibrate, experimental chronic renal failure, chronic kidney disease

## Abstract

Chronic kidney disease (CKD) is associated with elevated plasma fibrinogen concentration. However, the underlying molecular mechanism for elevated plasma fibrinogen concentration in CKD patients has not yet been clarified. We recently found that HNF1α was significantly upregulated in the liver of chronic renal failure (CRF) rats, an experimental model of CKD in patients. Given that the promoter region of the fibrinogen gene possesses potential binding sites for HNF1α, we hypothesized that the upregulation of HNF1α can increase fibrinogen gene expression and consequently plasma fibrinogen concentration in the experimental model of CKD. Here, we found the coordinated upregulation of *Aα*-chain fibrinogen and *Hnfα* gene expression in the liver and elevated plasma fibrinogen concentrations in CRF rats, compared with pair-fed and control animals. Liver Aα-chain fibrinogen and HNF1α mRNAs levels correlated positively with (a) liver and plasma fibrinogen levels and (b) liver HNF1α protein levels. The positive correlation between (a) liver Aα-chain fibrinogen mRNA level, (b) liver Aα-chain fibrinogen level, and (c) serum markers of renal function suggest that fibrinogen gene transcription is closely related to the progression of kidney disease. Knockdown of *Hnfα* in the HepG2 cell line by small interfering RNA (siRNA) led to a decrease in fibrinogen mRNA levels. Clofibrate, an anti-lipidemic drug that reduces plasma fibrinogen concentration in humans, decreased both HNF1α and Aα-chain fibrinogen mRNAs levels in (a) the liver of CRF rats and (b) HepG2 cells. The obtained results suggest that (a) an elevated level of liver HNF1α can play an important role in the upregulation of fibrinogen gene expression in the liver of CRF rats, leading to an elevated concentration of plasma fibrinogen, a protein related to the risk of cardiovascular disease in CKD patients, and (b) fibrates can decrease plasma fibrinogen concentration through inhibition of *HNF1α* gene expression.

## 1. Introduction

Cardiovascular disease (CVD) is the leading cause of mortality in patients with chronic kidney disease (CKD) [1,2]. Most patients with stage 3–4 CKD die of cardiovascular causes, rather than progress to the end-stage of kidney disease [3]. The main cause of cardiovascular diseases is atherosclerosis triggered in response to various insults that are very prevalent in CKD patients including (a) dyslipidemia, [3,4,5], (b) oxidative stress, (c) endothelial dysfunction, and (d) persistent inflammation [5,6,7,8]. Some studies have suggested that fibrinogen is also involved in the development of arteriosclerosis [9]. Fibrinogen, a six-chain molecule containing two copies of each of Aα, Bβ, and γ chains [10,11], is synthesized mainly in the liver and activated during the clotting process [12] and inflammation [13]. It has been postulated that fibrinogen can lead to an increase in cardiovascular risk because it (a) promotes platelet aggregation and fibrin formation and increases plasma viscosity and (b) participates in inflammatory processes [14]. Epidemiological studies in the general population have indicated that elevated plasma fibrinogen concentration is associated with an increased risk of cardiovascular events [15,16,17]. A very recently published review summarized data indicating that increased plasma fibrinogen concentrations are a risk factor for atherosclerotic cardiovascular diseases [18]. 

Numerous papers have reported that plasma fibrinogen concentration is also elevated at a different stage of CKD [19,20,21,22,23,24,25,26,27,28,29,30,31,32]; however, the molecular mechanism contributing to higher plasma fibrinogen concentrations in CKD patients is still unresolved. It has previously been shown that the increased fibrinogen synthesis rate contributes to the elevated concentration of plasma fibrinogen in CKD [33]. Thus, it is very likely that the elevated plasma fibrinogen concentrations in CKD patients result from increased liver fibrinogen gene transcription.

Based on the data indicating that (a) the promoter region of the fibrinogen gene possesses potential binding sites for HNF1α [34] and (b) HNF1α is significantly elevated in the liver of CRF rats [35,36,37], we hypothesize that this transcriptional factor can be involved in the upregulation of fibrinogen gene expression in the liver of rats with experimental CRF, which could, in turn, lead to the elevated level of plasma fibrinogen concentration. 

Thus, we examined the relationship between fibrinogen and *Hnfα* gene expression in the liver of CRF rats. To assess the direct impact of HNF1α on fibrinogen gene expression, we examined the effect of small interfering RNA (siRNA) on the expression of *HNF1α* and fibrinogen genes in the HepG2 cell line, which have optimal transfection efficiencies necessary for the siRNA methodology and introduces human relevance. We also studied the effect of clofibrate, an anti-lipidemic drug that lowers plasma fibrinogen concentration [38], on HNF1α mRNA and fibrinogen mRNA in the liver of CRF rats and in HepG2 cells.

The findings presented herein provide new information about the role of HNF1α in the upregulation of fibrinogen gene expression in CRF rats and suggest that the inhibition of *HNF1α* gene expression by clofibrate leads to a significant decrease in fibrinogen gene expression.

## 2. Results

### 2.1. Serum Concentrations of Markers of Renal Function in CRF Rats

**Figure 1:**
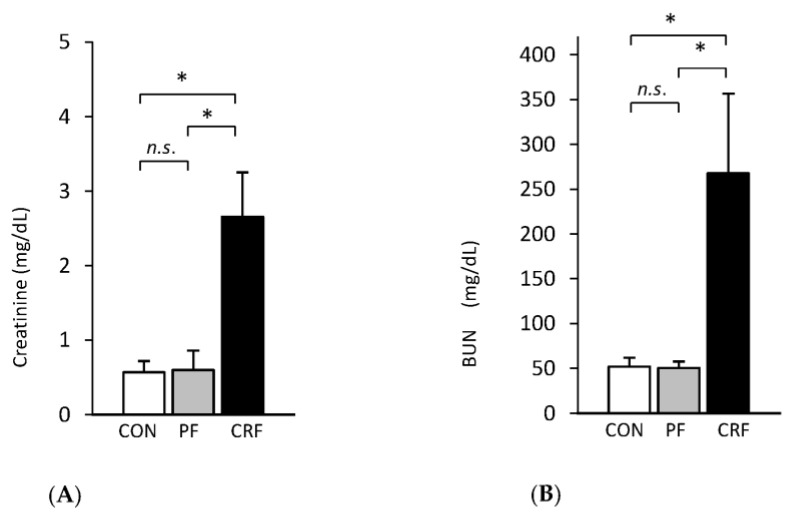
Serum concentrations of creatinine (**A**) and BUN (**B**) in: control (CON-□), pair-fed (PF-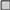), and chronic renal failure (CRF-■) rats. Graphs represent the mean ± SD from 10 controls, 10 pair-fed, and 10 chronic renal failure rats. Statistics: * *p* < 0.05, *n.s.*: not significant.

Concentrations of serum creatinine and blood urea nitrogen (BUN), which are markers of renal function, were approximately 4–5 times higher in CRF rats than in control (sham-operated) and pair-fed rats (Figure 1A,B). These results validated our experimental model and suggest that CRF induced by partial nephrectomy in rats correspond, at a rough estimate, to the late stage of CKD in patients.

### 2.2. Association between Hnfα and Fibrinogen Genes Expression in CRF Rats

**Figure 2:**
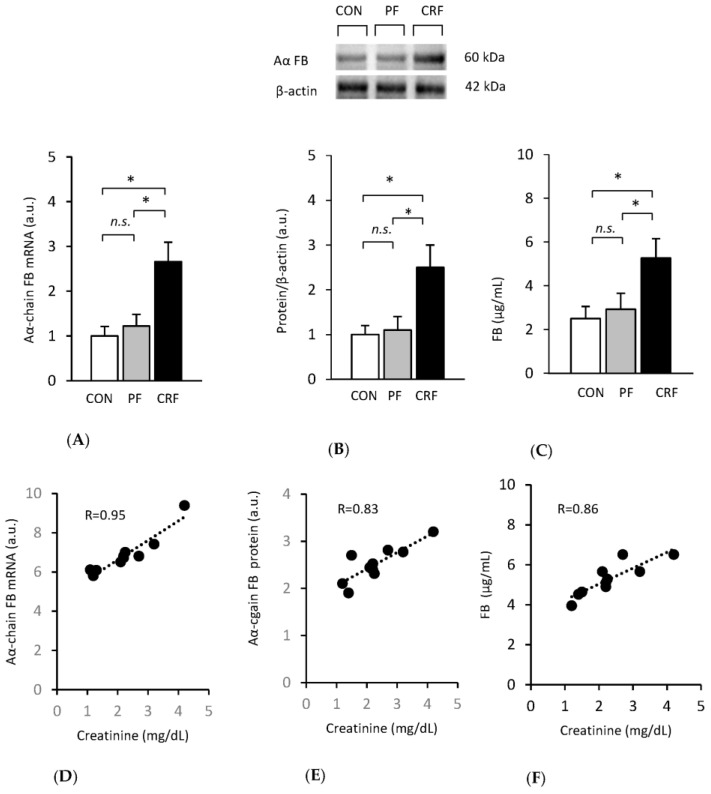
The increase in liver fibrinogen gene expression and its association with elevated plasma fibrinogen concentrations and serum concentrations of renal function markers in CRF rats. Aα-chain fibrinogen (FB) mRNA level (**A**). Representative Western blot analysis (top panel) and densitometric analysis of Western blot bands (bottom panel) of liver Aα-chain FB protein levels (**B**). Plasma fibrinogen (FB) concentrations (**C**) in control (CON, □), pair-fed (PF, 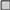), and chronic renal failure (CRF, ■) rats. Graphs represent the mean ± SD from 10 controls, 10 pair-fed rats, and 10 chronic renal failure rats. Statistics: * *p* < 0.05; *n.s.*: not significant. Correlation between serum creatinine concentrations and liver Aα-chain fibrinogen mRNA level in CRF rats (**D**). Serum creatinine concentrations and liver Aα-chain FB protein levels in CRF rats (**E**) and serum creatinine concentrations and plasma fibrinogen concentrations in CRF rats (**F**).

To determine the role of HNF1α in the regulation of fibrinogen gene expression in the liver of CRF rats, we began by examining the association between fibrinogen and *Hnfα* gene expression in the experimental model of CKD. Liver Aα-chain fibrinogen mRNA (Figure 2A) and protein (Figure 2B) levels were approximately twofold higher in the CRF rats than in the control or pair-fed rats. The intergroup differences in liver Aα-chain fibrinogen mRNA and protein levels determined by Western blot analysis were reflected by different plasma fibrinogen concentrations (Figure 2C).

Additionally, strong positive correlations between serum creatinine concentration and liver Aα-chain fibrinogen mRNA (Figure 2D) and liver fibrinogen (Figure 2E), as well as between serum creatinine concentration and plasma fibrinogen concentration (Figure 2F), were found. Essentially, similar associations between BUN concentration and liver Aα-chain fibrinogen mRNA and protein levels, as well as between BUN and plasma fibrinogen concentrations, were observed (not shown). The strong positive correlation between liver Aα-chain fibrinogen gene expression level (measured as fibrinogen mRNA and protein levels) and serum markers of renal function (serum creatinine and BUN concentrations) suggest that fibrinogen gene transcription is tightly related to the progression of kidney disease. Overall, the data presented in Figure 2 clearly indicate that in the experimental model of CKD, the increase in liver fibrinogen gene expression takes place, which is tightly associated with elevated plasma fibrinogen concentration and serum concentrations of renal function markers.

**Figure 3:**
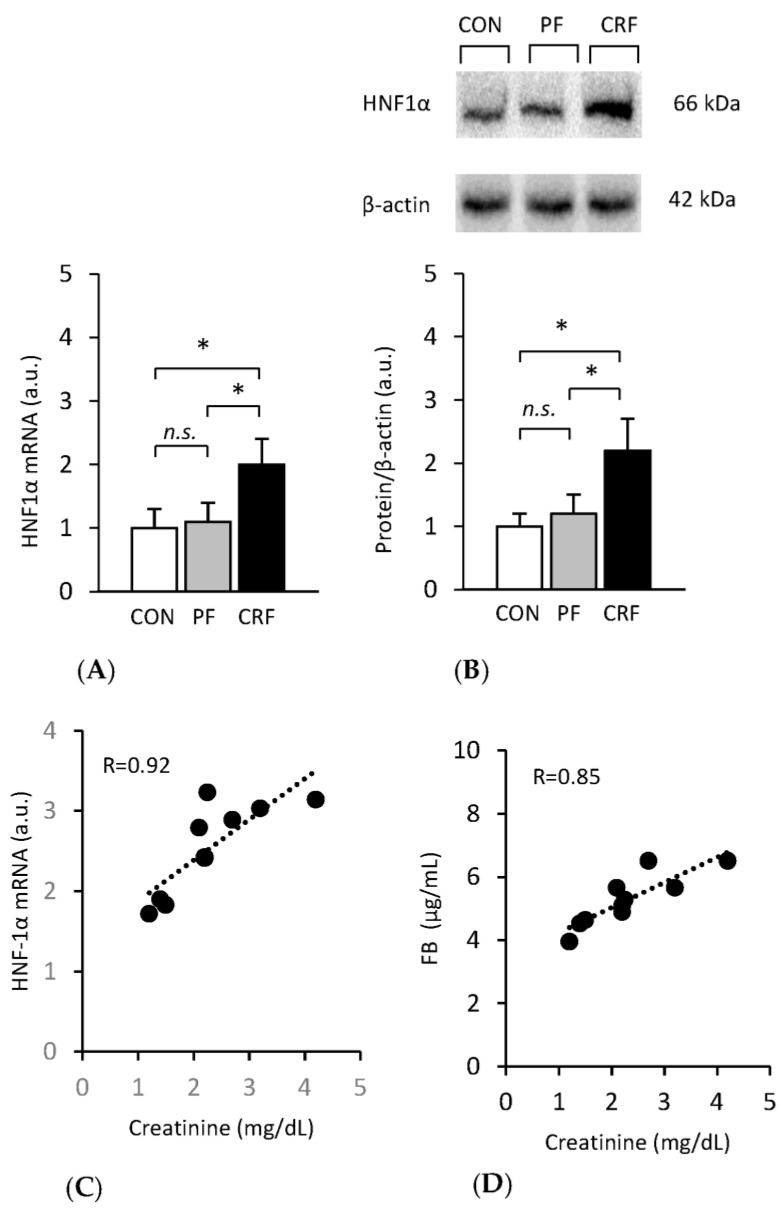
*Hnfα* gene expression in the liver of control, pair-fed, and CRF rats. Hnf1α mRNA levels in the liver (**A**). Representative Western blot analysis (top panel) and densitometric analysis of Western blot bands (bottom panel) of liver HNF1α protein levels (**B**) (β-actin was used as a standard). Control (CON, □), pair-fed (PF, 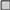), and chronic renal failure (CRF, ■) rats (means ± SDs). Correlation between serum creatinine concentrations and liver HNF-1 mRNA (**C**) and liver HNF1α protein levels (**D**). Statistics: * *p* < 0.05; *n.s.*: not significant.

As expected, the upregulation of liver fibrinogen gene expression in CRF rats was tightly associated with an increase in levels of liver HNF1α mRNA (Figure 3A) and liver HNF1α protein determined by Western blot analysis (Figure 3B). Again, strong positive correlations between serum creatinine concentration and liver HNF1α mRNA (Figure 3C), as well as between serum creatinine concentration and liver HNF1α protein levels, were found (Figure 3D). Strong positive correlations between BUN concentration and liver l HNF1α mRNA, as well as between BUN concentration and liver HNF1α protein levels, were also found (not shown). These results suggest that *hnf1α* gene expression increased in response to renal insufficiency.

Based on the data presented on Figure 2 and Figure 3, we calculated the relationship between liver Aα-chain fibrinogen and *Hnfα* gene expression. A strong positive correlation was found between (a) liver Aα-chain fibrinogen mRNA and liver HNF1α protein levels (*r* = 0.83; *p* < 0.001), (b) liver Aα-chain protein and liver HNF1α protein levels (*r* = 0.79; *p* < 0.001), and (c) plasma fibrinogen concentration and liver HNF1α protein (*r* = 0.74; *p* < 0.001) levels.

Together, the results presented in Figure 2 and Figure 3 suggest that overproduction (due to overexpression) of HNF1α promoted the expression of the Aα-chain fibrinogen gene in CRF rats, consequently leading to the increase in plasma fibrinogen concentration.

### 2.3. Silencing of HNF1α Gene Expression by siRNA Decreases Aα-Chain Fibrinogen mRNA Level

**Figure 4:**
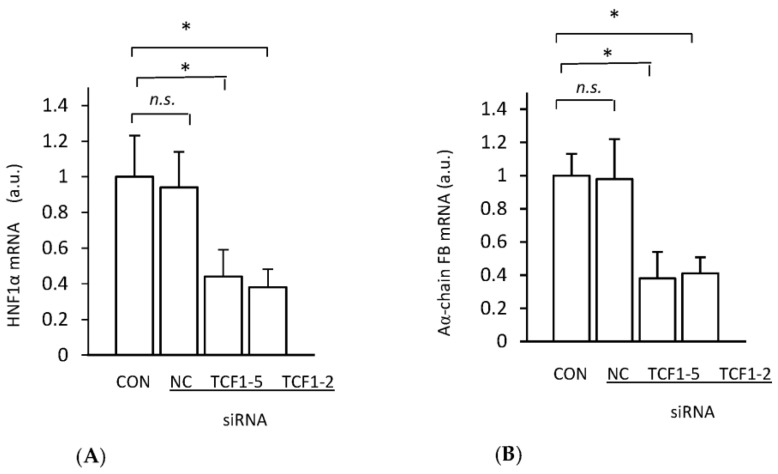
HNF1α mRNA levels (**A**) and Aα-chain fibrinogen (FB) mRNA levels (**B**) in lipofectamine-treated HepG2 cells (CON) and HepG2 cells transfected with two different siRNA targeting HNF1α (TCF1-2 or TCF1-5). NC represents negative control. Graphs represent the mean ± SD of results from six plates performed in three different experiments. Statistics: * *p* < 0.05; *n.s.*: not significant.

To verify the hypothesis that HNF1α is a transcriptional factor directly involved in the upregulation of fibrinogen gene expression in the liver of CRF rats, we assessed the deregulation of *HNF1α* gene expression in HepG2 cells by silencing its expression with small interfering RNA (siRNA). The knockdown of the endogenous *HNF1α* expression, measured as HNF1α mRNA (Figure 4A), by two different siRNAs in HepG2 cells was accompanied by a decrease in Aα-chain fibrinogen mRNA level (Figure 4B).

These results indicate that *HNF1α* gene expression is indeed engaged in the regulation of liver fibrinogen gene expression. Thus, one can assume that the upregulation of *Hnfα* gene expression is directly related to the upregulation of fibrinogen gene expression, which leads to increased plasma fibrinogen concentration in CRF rats.

### 2.4. Clofibrate Decreases Aα-Chain Fibrinogen MRNA Level In Vivo and In Vitro

**Figure 5:**
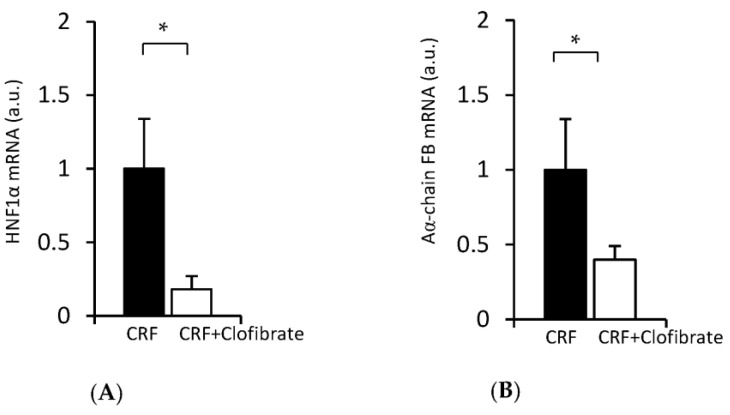
HNF1α mRNA level (**A**) and Aα-chain fibrinogen (FB) mRNA levels (**B**) in the liver of untreated (CRF, ■) and clofibrate-treated (CRF + Clofibrate, □) rats. Graphs represent the mean ± SD of results from 10 untreated or 10 clofibrate-treated CRF rats. * *p* < 0.05.

Clinical studies have shown that fibrates, also known as anti-lipemic drugs, reduce plasma fibrinogen concentrations [38]. Considering that (a) the plasma fibrinogen concentration largely depends on the factor(s) regulating fibrinogen biosynthesis in the liver [33,39] and (b) clofibrate reduces the HNF1α mRNA level in the liver [35,36] of CRF rats, we hypothesized that fibrates can decrease the biosynthesis of fibrinogen and consequently plasma fibrinogen concentration through the inhibition of *HNF1α* gene expression. To verify this hypothesis, we examined the effect of clofibrate on HNF1α mRNA and Aα-chain fibrinogen mRNA in CRF rats treated with clofibrate. The results presented in Figure 5 indicate that clofibrate coordinately decreased HNF1α mRNA (Figure 5A) and Aα-chain fibrinogen mRNA (Figure 5B) levels in the liver of CRF rats treated with clofibrate.

**Figure 6:**
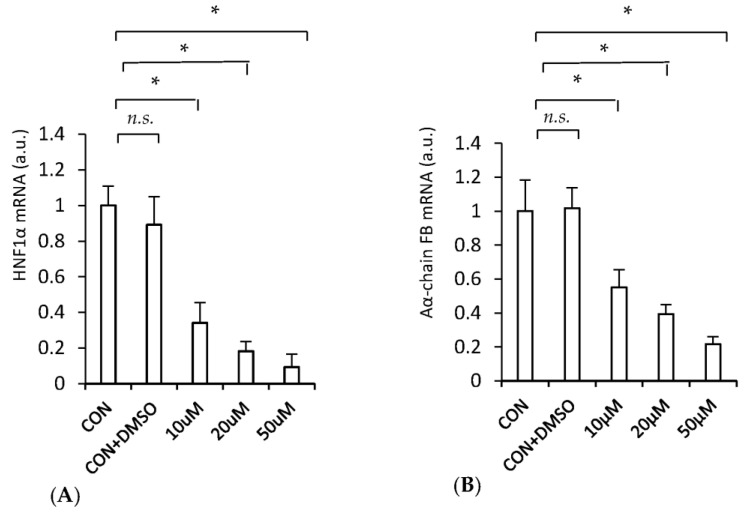
HNF1α mRNA levels (**A**) and Aα-chain fibrinogen (FB) mRNA levels (**B**) in HepG2. CON: control HepG2 untreated cells; CON + DMSO: control HepG2 cells treated with 0.5% DMSO. HepG2 cells treated with 10, 20, 50 µM clofibrate dissolved in 0.5% DMSO. Graphs represent the mean ± SD of results from six plates performed in three different experiments. Statistics: * *p* < 0.05; *n.s.*: not significant.

To confirm that the in vivo effect of clofibrate on HNF1α and fibrinogen mRNAs (Figure 5) is due to the direct effect of the drug on hepatocytes (but is not the result of lowered serum lipid concentrations or another unknown mechanism), we examined the effect of clofibrate on HNF1α and Aα-chain fibrinogen mRNAs in HepG2 cells. As shown in Figure 6, clofibrate in a dose-dependent manner decreased the HNF1α mRNA level (Figure 6A). This was paralleled by the decrease in Aα-chain fibrinogen mRNA (Figure 6B).

Thus, the results presented in Figure 5 (showing the in vivo study) and Figure 6 (showing the in vitro study) further confirm the view that HNF1α may play an important role in the regulation of fibrinogen biosynthesis (via increased fibrinogen gene transcription) and consequently in plasma fibrinogen concentrations in the experimental model of CKD.

## 3. Discussion

The main purpose of the present study was to explore the potential role of HNF1α in the regulation of fibrinogen gene expression in the liver of rats with CRF. HNF1α (also known as transcription factor 1, TCF1) binds as a dimer to target genes and regulates as a member of the liver transcription factor the biosynthesis of proteins that participate in a wide range of hepatocellular biochemical functions [40]. Accordingly, HNF1α potential binding sites have been identified in many genes involved in different biological functions, including the fibrinogen gene [34]. Recently, we found that HNF1α was significantly upregulated in the liver of CRF rats [35,36]. Thus, we hypothesized that upregulation of *Hnfα* gene expression in CRF rats may play a key role in upregulation of the transcriptional activity of the fibrinogen gene and consequently in the increase in plasma fibrinogen concentration, which, in turn, may promote cardiovascular disease (CVD) [16,17,18].

In agreement with our hypothesis, the major and original finding of this study was that the upregulation of *Hnfα* gene expression in the liver of rats (i.e., the experimental model of CKD in humans) is tightly associated with the upregulation of fibrinogen gene expression (measured as liver mRNA and protein level) and elevated plasma fibrinogen concentration. This suggests that HNF1α as a transcriptional factor is involved in the upregulation of fibrinogen gene expression in the liver of rats with CRF. This was confirmed by (a) the strong positive correlation between Aα-chain fibrinogen gene expression (measured as liver Aα-chain fibrinogen mRNA and protein levels as well as plasma fibrinogen concentration) and *Hnfα* gene expression (determined as mRNA and liver protein levels) in CRF rats; (b) the coordinated reduction in the expression of fibrinogen and *Hnfα* genes caused by clofibrate in vivo (in CRF rats treated with clofibrate) and in vitro (in the HepG2 cell line); (c) the decrease in fibrinogen mRNA level due to a *HNF1α* gene expression knockdown with siRNA in HepG2 cells. Moreover, the fibrinogen gene possesses HNF1α potential binding sites in its promoter [34]. Therefore, the activation of fibrinogen gene transcription in the liver of CRF rats could result in HNF1α binding to the fibrinogen gene. Overall, our studies show for the first time that the upregulation of *Hnfα* gene expression in CRF rats contributes to fibrinogen gene expression and consequently to the elevated level of plasma fibrinogen. Given the coordinated regulation of the expression of fibrinogen and *HNF1α* genes in the HepG2 cell line (human liver cells), one can suppose that HNF1α also contributes to the overexpression of the fibrinogen gene in the liver of CKD patients. Therefore, it appears plausible that pharmacological approaches to block HNF1α upregulation may be beneficial for decreased plasma fibrinogen concentration and consequently for reduced atherosclerotic cardiovascular diseases risk. Due to the obvious limitation (availability of human tissue, especially liver), most of the studies presented here cannot be performed in patients with CKD. However, the results presented herein and regarding the elevated fibrinogen concentration in the plasma of CKD patients are confirmed by several clinical studies [41,42], and we can hypothesize that processes similar to those shown in CRF rats may be found in CKD patients. However, further research is required to confirm the role of HNF1α in upregulation of the fibrinogen gene in humans.

Although the molecular mechanism underlying the upregulation of *Hnfα* gene expression in CRF rats is still unknown, it is tempting to speculate that persistent inflammation plays an important role in this process. Accordingly, we postulate that chronic inflammation associated with CRF leads to upregulation of HNF1α, which, in turn, activates fibrinogen gene expression. This leads to an increase in plasma fibrinogen concentration, which is a risk factor for CVD [18]. Thus, the results presented here and reported previously suggest that HNF1α is a master transcription regulator of genes encoding for (a) fibrinogen (results presented herein), (b) proteins involved in lipid metabolism [35], and (c) proinflammatory proteins [36] in chronic kidney disease. Collectively, the abovementioned data suggest an important role for the overexpression of HNF1 in the development of dyslipidemia and inflammation and consequently in the development of atherosclerosis in CKD patients.

Special attention should be paid to the effect of clofibrate on fibrinogen gene expression in CRF rats. First, coordinated downregulation of HNF1α and fibrinogen both in vivo and in vitro by clofibrate suggests that HNF1α plays a crucial role in fibrinogen gene expression. Second, the results presented in this paper may explain, at least in part, the molecular mechanism of fibrates’ action on plasma fibrinogen concentration. Clinical studies have shown that fibrates, which are lipid-lowering drugs, and peroxisome proliferator-activated receptor-α (PPAR α) agonists are also very effective in reducing plasma fibrinogen concentration [38]. It has been proposed that fibrates suppress fibrinogen gene expression in rodents via the activation of PPARα; however, the exact molecular mechanism by which PPARα acts is not well understood [43]. The data presented here, both in vivo (Figure 5) and in vitro (Figure 6), suggest that fibrates are able to decrease liver fibrinogen gene expression through the inhibition of *HNF1α* gene expression, which leads to decreased plasma fibrinogen concentration. It should be noted that transcriptional suppression of *HNF4* in the liver of rats treated with bezafibrate was also reported by Hertz et al. [44]. Therefore, the above results and those published by Hertz et al. [44] suggest that both *HNF1α* and *HNF4* genes are downregulated by fibrates, leading to the suppression of target genes including fibrinogen (data presented here) and *apo CIII* [44] or transferrin [45] genes.

From a practical point of view, fibrates constitute an important group of drugs for the treatment of dyslipidemia. They lower blood triacylglycerols and LDL cholesterol concentration while increasing HDL cholesterol [46]. Fibrates have also been effective drugs for reducing cardiovascular disease in patients with mild to moderate renal insufficiency [47]. However, the effectiveness of fibrates used to treat dyslipidemia in CKD patients was limited due to the reduction in the glomerular filtration rate at the beginning of treatment. Nevertheless, numerous published data have indicated that fibrates improve the serum lipid profile in CKD patients [47,48,49]. Some authors have suggested that the modest increase in serum creatinine concentration (especially at the beginning of treatment) cannot be a limiting factor for the treatment with fibrates of patients with mild CKD [47]. Moreover, recently published results indicated that pemafibrate, a novel selective PPARα activator, is a good and safe drug for treating serum lipid abnormalities in patients with CKD [50]. It is likely, therefore, that downregulation of fibrinogen gene expression by fibrates, used to treat dyslipidemia in CKD patients, may have potential practical significance.

It is clear that we cannot exclude other mechanisms (besides inhibition of *HNF1α* gene expression) involved in the regulation of fibrinogen gene expression by clofibrate. Hertz et al. reported that the suppression of *apo CIII* [44] and transferrin [45] genes by fibrates is due to the displacement of HNF4 from the apo CIII promoter or transferrin promoter by PPARα-RXR. As HNF1α and HNF4α physically interact [51] and transcriptionally antagonize [51] or synergize [52] each other, it is very likely that the displacement of HNF1α (transcriptional activator) from the fibrinogen promoter exerted by clofibrate bound to PPARα (which form PPARα-RXR complex) may also contribute to the inhibition of fibrinogen gene expression. However, further studies are necessary to confirm this mechanism.

It is generally believed that the cardioprotective effect of fibrates is associated with decreasing (a) circulating lipids (total cholesterol, LDL cholesterol, and triacylglyceroles), (b) vascular cell adhesion molecules 1 (VCAM-1), (c) intercellular cell adhesion molecules 1 (ICAM-1), (d) C-reactive protein (CRP), (e) monocyte chemoattractant protein-1 (MCP-1), (f) interleukin-6 (Il-6), (g) tumor necrosis factor α (TNF-α), and (h) fibrinogen and PAI-1 [53]. The results presented here indicated that fibrates also may improve blood coagulation and fibrinolytic activity by lowering the plasma fibrinogen concentration. Therefore, the reduction in fibrinogen gene expression via the inhibition of *HNF1α* gene expression (or by another mechanism), and consequently the decrease in plasma fibrinogen concentration by clofibrate, is among the potential molecular mechanisms underlying the cardioprotective effect of fibrates. Interestingly, PPARα negatively regulates proinflammatory signaling pathways [54]. The presented results significantly extend our previous observation showing that HNF1α regulates genes, the products of which are involved in the regulation of lipids [35] as well as CRP and Il-6 [36] in CRF rats.

## 4. Materials and Methods

### 4.1. Animals

The study was performed using 10-week-old male Wistar rats weighing approximately 250 g at the beginning of the experiment (before induction of CRF). All experiments were conducted according to our institutional guidelines for the care and use of laboratory animals.

### 4.2. CRF Rats—Experimental Model of CKD

CRF was induced by subtotal nephrectomy as described previously [55]. Sham-operated animals served as the control. All animals (10 rats in each group, i.e., CRF; sham-operated and pair-fed rats) were kept in individual wire-mesh cages and allowed free access to tap water. CRF and sham-operated rats were allowed free access to a commercial diet that has been previously described [56]. Pair-fed rats received daily the amount of food corresponding to that consumed by CRF animals. The air temperature in the animal room was set at 22 °C and the lighting schedule was controlled (12 h light/dark cycles). Six weeks after induction of CRF (between 8.00 and 10.00 am): (a) blood samples (for plasma or serum isolation) from abdominal aorta and (b) pieces (approximately 0.5 g) of liver were collected under thiopental anesthesia. Then, rats were euthanized. Pieces of liver were immediately frozen in liquid nitrogen and then stored at −80 °C until the expression of the studied genes was determined. Plasma or serum was obtained after blood centrifugation at 1500× *g* for 10 min.

### 4.3. Clofibrate Treatment of CRF Rat

Five weeks after the induction of CRF, the rats were given clofibrate (250 mg/kg of body weight for seven successive days) as described previously [57].

### 4.4. HepG2 Cell Culture

HepG2 cells (a human hepatocellular carcinoma cell line) were obtained from ATCC (Manassas, VA, USA). Cells were maintained in standard Minimum Essential Eagle’s Medium (MEM: 5650; Sigma-Aldrich, St. Louis, MO, USA) supplemented with: 2 mM glutamine, 10% fetal bovine serum, penicillin (100 IU per mL), and streptomycin (100 µg per mL) at 37 °C under a humidified 95%/5% (*v*/*v*) mixture of air and CO_2_. Two days before the main experiments, HepG2 cells were passaged in 6-well plates at 10 × 10^−4^ cells per well. Then, cells were cultured and grown to approximately 70% confluence.

### 4.5. Clofibrate Treatment of HepG2 Cells

Clofibrate (Merck & Co., Inc., Whitehouse Station, NJ, USA) was dissolved in dimethylsulfoxide (DMSO) and added to the cell culture at final concentrations of 10 µM, 20 µM, and 50 µM. Control cells were run in parallel, with a respective concentration of DMSO. After 48 h of incubation, the cells were washed twice with 1 mL of PBS and used for RNA isolation.

### 4.6. Small Interfering RNA (siRNA) Transfection

Two different sequences of siRNA targeting HNF-1α were used: (a) Hs -TCF1–2, no SI00011620 and (b) Hs -TCF1-5, no SI03095015. AllStars Negative Control, no 1027280 was used as the negative control (siRNA NC). siRNAs were obtained from Qiagen (Crawley, UK). HepG2 cells treated by lipofectamine were used as controls (CON). HepG2 cells were transfected with siRNA at concentrations of 10 nM using 0.1% (*v*/*v*) Lipofectamine RNA iMAX (Invitrogen, Paisley, UK), as described in the manufacturer′s protocol. We had to use the HepG2 cell line because HepG2 has a high transfection efficiency necessary for the siRNA methodology and the use of this human cell line was a way to introduce elements of human metabolism in the presented work. Transfection was performed in serum-free OptiMEM (Invitrogen, Paisley, UK). Cells were harvested after 48 h and used for total RNA or protein extraction.

### 4.7. Liver and HepG2 Cells RNA Isolation

Total RNA was extracted from the frozen liver using the guanidinium isothiocyanate-phenol/chloroform method [58]. The GenElute™ Mammalian Miniprep Kit (Sigma-Aldrich, St. Louis, MO, USA) was used for isolation of total RNA from HepG2 cells. The obtained RNA concentration was determined from the absorbance at 260 nm. All obtained samples had a 260/280 nm absorbance ratio of about 2.0.

### 4.8. CDNA Synthesis

First-strand cDNA was synthesized from 1 µg of total RNA (RevertAid First Strand cDNA Synthesis Kit (Thermo Fisher Scientific Baltics UAB V. A., Vilnius, Lithuania). Prior to amplification of cDNA, each RNA sample was treated with RNase-free Dnase I (Thermo Fisher Scientific Baltics UAB V. A., Vilnius, Lithuania), at 37 °C for 30 min.

### 4.9. Determination of HNF1α and Aα-Fibrinogen mRNA Level by RT-PCR

Rat Aα-fibrinogen, HNF1α, β-actin, and TBP (TATA-box binding protein) mRNA levels were quantified by RT-PCR using a Chromo4 Real Time Detection System (Bio-Rad Laboratories, Inc., Hercules, CA, USA). Primers were designed with the Sequence Analysis software package (Informagen, Inc. Newington, NH, USA) from gene sequences obtained from the Ensembl Genome Browser (www.ensembl.org, accessed on 16 January 2020). The rat sequences of primer pairs (sense and antisense) used in this study were: (a) 5′-AAGATGACACGGATGACGATGG-3′ (sense) and 5′-GGTTGAGACCCGTAGTGTCC-3′ (antisense) for the HNF1α; (b) 5′-AAATGTGCAGGTGTTGACCA-3′ (sense) and 5′-CACGCTCCTCCTGAAGAATC-3′ (antisense) for the fibrinogen; (c) 5′-TGTCACCAACTGGGACGATA-3′ (sense) and 5′-GGGGTGTTGAAGGTCTCAAA-3′ (antisense) for β-actin; (d) 5′-CACCGTGAATCTTGGCTGTAAAC-3′ (sense) and 5′-ATGATGACTGCAGCAAACCG-3′ (antisense) for the *Tbp.*

Primers for human: (a) HNF1α (qHsaCED0001918), (b) fibrinogen (qHsaCED0046822), β-actin (qHsaCED0036269), and c) TBP (qHsaCID0007122) assayed in HepG2 cells, were obtained from Bio-Rad Laboratories, Inc., USA. Real-time PCR amplification was performed in 20 μL volumes using iQ SYBR Green Supermix (Bio-Rad Laboratories, Hercules, CA, USA). Each reaction contained cDNA and 0.3 μM of each primer. Control reactions, with omission of the RT step or with no template cDNA added, were performed with each assay. All samples were run in triplicate. To compensate for variations in the amount of added RNA and in the efficiency of the reverse transcription, β-actin or TBP mRNA was quantified in the corresponding samples and the results were normalized to these values. It should be noted that results obtained with β-actin and TBP (as internal standards) were similar. The relative quantities of transcripts were calculated using the 2^−ΔΔCT^ formula [59]. The results are expressed in arbitrary units, with one unit representing the mean mRNA level determined in a control group. Amplification of specific transcripts was confirmed by obtaining the melting curve profiles and subjecting the amplification products to agarose gel electrophoresis.

### 4.10. Determination of Plasma Fibrinogen Concentration in CRF Rats

Commercially available ELISA kits were used to estimate plasma fibrinogen (QAYEE-BIO For Life Science, Shanghai, China) concentrations.

### 4.11. Western Blot Analysis of HNF1α, Aα-Chain FB, and β-Actin in Rat Liver

Frozen liver samples were thawed; minced finely with scissors; homogenized in a buffer containing: 10 mM Tris–HCl (pH 7.8), 2% SDS, 10 mM DTT, and proteinase inhibitors (Sigma); centrifuged at 15,000× *g* for 20 min at 20 °C. Supernatants were collected and the protein concentration was determined by the Bradford assay. Supernatants containing 20 μg of total protein were separated by 10% SDS-PAGE and electroblotted onto Immobilon Transfer Membrane (Millipore). The following antibodies were used: monoclonal antibody against HNF1 (sc-393925), monoclonal antibody against Fibrinogen α (sc-398806), and polyclonal antibody against Actin (sc-7210), all from Santa Cruz Biotechnology Inc. (Dallas, TX, USA). HRP-conjugated secondary antibodies (sc-2030 and sc-2004) were obtained from Santa Cruz Biotechnology and the HAF019 from R&D Systems. Immunodetection was accomplished with enhanced chemiluminescence using Western blotting Luminol Reagent (sc-2048, Santa Cruz Biotechnology).

### 4.12. Serum Creatinine and Blood Urea Nitrogen (BUN) Concentration

Serum creatinine and BUN concentrations were determined using a Hitachi 704 auto analyzer.

### 4.13. Statistics

The statistical significance of differences between groups was assessed by one-way analysis of variance (ANOVA) followed by Student’s *t*-test and one-way analysis of variance (ANOVA) followed by Tukey’s post hoc test. The Sigma Stat software was used. The results are presented as means ± SDs. Differences between groups were considered significant when *p* < 0.05. The relations between two variables were calculated using the Pearson’s correlation.

## 5. Conclusions

The results presented in this paper indicate that the increased HNF1α in the liver of CRF rats results in increased fibrinogen gene expression. This consequently leads to increased circulating fibrinogen concentration. Perhaps the increase in plasma fibrinogen concentration is one of the factors that increase the risk of CVD in the course of chronic kidney disease. Moreover, we have shown that clofibrate is able to decrease liver fibrinogen gene expression via inhibition of *HNF1α* gene expression, which leads to the decrease in plasma fibrinogen concentration.

## Data Availability

Not applicable.
